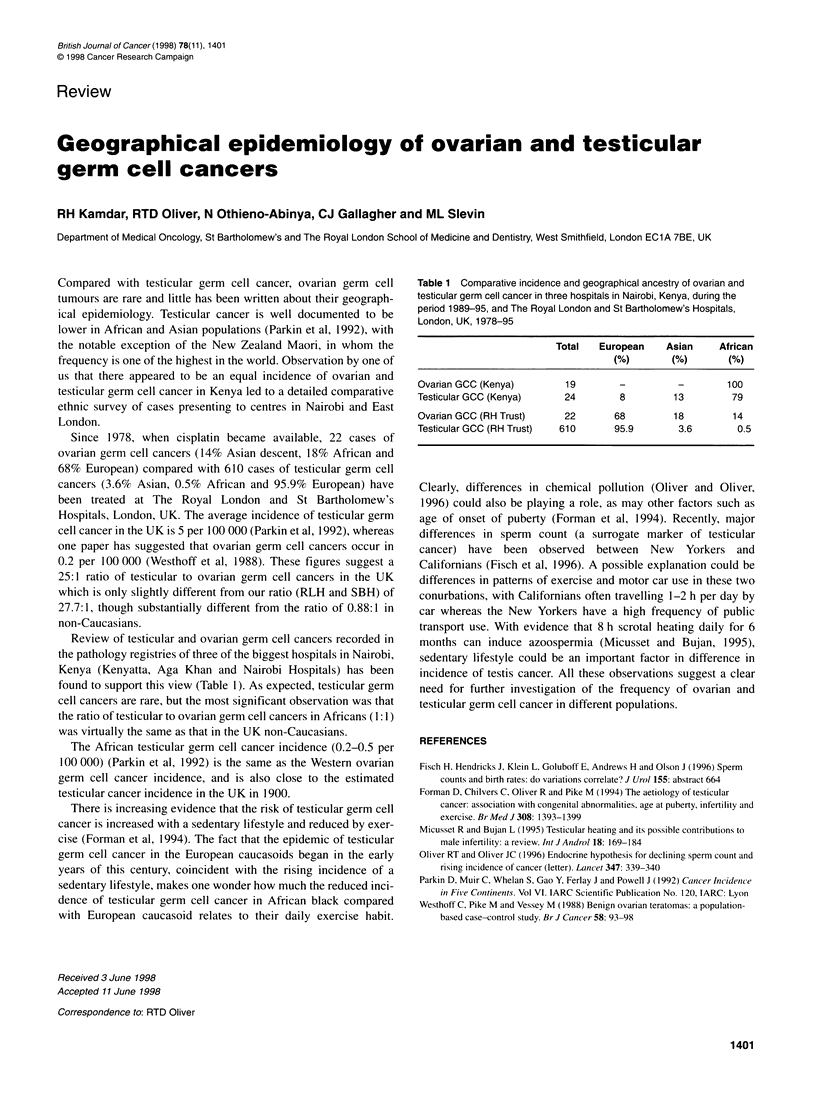# Geographical epidemiology of ovarian and testicular germ cell cancers.

**DOI:** 10.1038/bjc.1998.697

**Published:** 1998-12

**Authors:** R. H. Kamdar, R. T. Oliver, N. Othieno-Abinya, C. J. Gallagher, M. L. Slevin

**Affiliations:** Department of Medical Oncology, St Bartholomew's and The Royal London School of Medicine and Dentistry, West Smithfield, UK.


British Journal of Cancer (1998) 78(11), 1401
© 1998 Cancer Research Campaign

Review

### Geographical epidemiology of ovarian and testicular germ cell cancers

**RH Kamdar, RTD Oliver, N Othieno-Abinya, CJ Gallagher and ML Slevin**

Department of Medical Oncology, St Bartholomew's and The Royal London School of Medicine and Dentistry, West Smithfield, London EC1A 7BE, UK

Compared with testicular germ cell cancer, ovarian germ cell tumours are rare and little has been written about their geographical epidemiology. Testicular cancer is well documented to be lower in African and Asian populations (Parkin et al, 1992), with the notable exception of the New Zealand Maori, in whom the frequency is one of the highest in the world. Observation by one of us that there appeared to be an equal incidence of ovarian and testicular germ cell cancer in Kenya led to a detailed comparative ethnic survey of cases presenting to centres in Nairobi and East London.

Since 1978, when cisplatin became available, 22 cases of ovarian germ cell cancers (14% Asian descent, 18% African and 68% European) compared with 610 cases of testicular germ cell cancers (3.6% Asian, 0.5% African and 95.9% European) have been treated at The Royal London and St Bartholomew's Hospitals, London, UK. The average incidence of testicular germ cell cancer in the UK is 5 per 100 000 (Parkin et al, 1992), whereas one paper has suggested that ovarian germ cell cancers occur in 0.2 per 100 000 (Westhoff et al, 1988). These figures suggest a 25:1 ratio of testicular to ovarian germ cell cancers in the UK which is only slightly different from our ratio (RLH and SBH) of 27.7:1, though substantially different from the ratio of 0.88:1 in non-Caucasians.

Review of testicular and ovarian germ cell cancers recorded in the pathology registries of three of the biggest hospitals in Nairobi, Kenya (Kenyatta, Aga Khan and Nairobi Hospitals) has been found to support this view (Table 1). As expected, testicular germ cell cancers are rare, but the most significant observation was that the ratio of testicular to ovarian germ cell cancers in Africans (1:1) was virtually the same as that in the UK non-Caucasians.

The African testicular germ cell cancer incidence (0.2–0.5 per 100 000) (Parkin et al, 1992) is the same as the Western ovarian germ cell cancer incidence, and is also close to the estimated testicular cancer incidence in the UK in 1900.

There is increasing evidence that the risk of testicular germ cell cancer is increased with a sedentary lifestyle and reduced by exercise (Forman et al, 1994). The fact that the epidemic of testicular germ cell cancer in the European caucasoids began in the early years of this century, coincident with the rising incidence of a sedentary lifestyle, makes one wonder how much the reduced incidence of testicular germ cell cancer in African black compared with European caucasoid relates to their daily exercise habit.

**Table 1** Comparative incidence and geographical ancestry of ovarian and testicular germ cell cancer in three hospitals in Nairobi, Kenya, during the period 1989–95, and The Royal London and St Bartholomew's Hospitals, London, UK, 1978–95

| | Total | European (%) | Asian (%) | African (%) |
|---|---|---|---|---|
| Ovarian GCC (Kenya) | 19 | – | – | 100 |
| Testicular GCC (Kenya) | 24 | 8 | 13 | 79 |
| Ovarian GCC (RH Trust) | 22 | 68 | 18 | 14 |
| Testicular GCC (RH Trust) | 610 | 95.9 | 3.6 | 0.5 |

Clearly, differences in chemical pollution (Oliver and Oliver, 1996) could also be playing a role, as may other factors such as age of onset of puberty (Forman et al, 1994). Recently, major differences in sperm count (a surrogate marker of testicular cancer) have been observed between New Yorkers and Californians (Fisch et al, 1996). A possible explanation could be differences in patterns of exercise and motor car use in these two conurbations, with Californians often travelling 1–2 h per day by car whereas the New Yorkers have a high frequency of public transport use. With evidence that 8 h scrotal heating daily for 6 months can induce azoospermia (Micusset and Bujan, 1995), sedentary lifestyle could be an important factor in difference in incidence of testis cancer. All these observations suggest a clear need for further investigation of the frequency of ovarian and testicular germ cell cancer in different populations.

### REFERENCES

Fisch H, Hendricks J, Klein L, Goluboff E, Andrews H and Olson J (1996) Sperm counts and birth rates: do variations correlate? *J Urol* **155**: abstract 664

Forman D, Chilvers C, Oliver R and Pike M (1994) The aetiology of testicular cancer: association with congenital abnormalities, age at puberty, infertility and exercise. *Br Med J* **308**: 1393–1399

Micusset R and Bujan L (1995) Testicular heating and its possible contributions to male infertility: a review. *Int J Androl* **18**: 169–184

Oliver RT and Oliver JC (1996) Endocrine hypothesis for declining sperm count and rising incidence of cancer (letter). *Lancet* **347**: 339–340

Parkin D, Muir C, Whelan S, Gao Y, Ferlay J and Powell J (1992) *Cancer Incidence in Five Continents*. Vol VI. IARC Scientific Publication No. 120, IARC: Lyon

Westhoff C, Pike M and Vessey M (1988) Benign ovarian teratomas: a population-based case–control study. *Br J Cancer* **58**: 93–98

*Received 3 June 1998*
*Accepted 11 June 1998*

*Correspondence to:* RTD Oliver